# Endometriosis still a challenge

**Published:** 2014-09-25

**Authors:** C Mehedintu, MN Plotogea, S Ionescu, M Antonovici

**Affiliations:** "Nicolae Malaxa" Clinical Hospital

**Keywords:** endometriosis, fertility, pain, dysmenorrhea, endometriosis therapy

## Abstract

Abstract

Endometriosis is a debilitating disease with features of chronic inflammation. Endometriosis appears to be one of the most common benign gynecological proliferations in premenopausal women since it is estimated that 10–15% of reproductive aged women suffer from pelvic endometriosis. The biology of endometriosis is unclear. Despite its prevalence, this disease remains poorly understood and current studies prove that there is no relationship between the extent of the disease and its symptomatology. There is no blood test available for the diagnosis of endometriosis. Up to this point, there is no single very successful option for the treatment of endometriosis. Due to the relatively poor efficacy of hormonal therapy for endometriosis, several other experimental therapies are currently undergoing clinical trial.

## Introduction

Endometriosis is a debilitating disease with features of chronic inflammation and is defined as the presence of functional endometrial glands and stroma outside the uterine cavity, the most common locations for the ectopic endometrial implants being the ovaries, the fossa ovarica, the uterosacral ligaments and the posterior cul-de-sac [**1**]. Endometriosis is similar to malignancies in some ways: progressive and invasive growth, estrogen-dependent growth, recurrence and a tendency to metastasize [**2,3**]. This disease has been classified in four stages based on the severity, amount, location, depth and size of growths, those stages being: stage I (minimal disease), stage II (mild disease), stage III (moderate disease) and stage IV (severe disease) [**1,4**]. The notion of deep endometriosis implies endometriosis infiltrating deeper than 5 mm under the peritoneum [**5**]. This classification however is unsuccessful in predicting the clinical outcomes, including the symptomatology, respectively the pain [**6**]. This being said, medical professionals dealing with endometriosis face a lot of problems in diagnosis, treatment and follow up of patients. Endometriosis appears to be one of the most common benign gynecological proliferations in premenopausal women since it is estimated that 10–15% of reproductive aged women suffer from pelvic endometriosis [**7**]. Being one of the most common benign gynecological conditions, endometriosis is a debilitating disease with detrimental effects on social, occupational and psychological functioning. The prevalence of this disease increases up to 30% in patients with infertility and up to 45% in patients with chronic pelvic pain [**8**]. Etiology and etiopathogeny of endometriosis The biology of endometriosis is unclear. While the etiology of endometriosis still remains unclear, the mechanism most widely accepted for the development of the peritoneal endometriotic lesions is via retrograde menstruation [**9**]. The other suggested mechanisms would be coelomic metaplasia, immune system abnormalities, genetic causes, environmental and lifestyle factors. It is possible that several of these factors play a role or those specific subtypes of disease are due to specific underlying biological pathways. The research of these mechanisms is far from coming to an end. Endometriosis develops in women of reproductive age and regresses after menopause or after ovariectomy [**10**] suggesting that the establishment and growth of ectopic implants is dependent on ovarian steroids, similarly to eutopic endometrium. Retrograde Menstruation In Sampson’s opinion, during normal menstruation, menstrual debris including viable eutopic endometrial cells, growth factors and cytokines could travel in a retrograde way through the fallopian tubes into the pelvic cavity, where these cells would be able to invade and proliferate on surrounding tissues [**11**]. This theory is supported by the histological similarity between endometriotic tissue and normal endometrial tissue, also by the frequent localization of endometrial lesions on the organs situated in the pelvic cavity. The finding that retrograde menstruation frequently occurs in menstruated women also supports this hypothesis. It has been demonstrated that forced surgical induction of retrograde menstruation in primate models conducted to the development of endometriosis in 50% of cases [**12**]. However, menstrual debris is present in the peritoneal cavity of up to 90% of women at the reproductive age. While retrograde menstruation occurs in 90% of the menstruating women, endometriosis is estimated to affect only 1 in 10 of these women, therefore possible explanations for the adhesion and growth of endometriotic lesions may include other factors like increased exposure to menstrual debris (increased menstrual flow, shorter cycle length), abnormal eutopic endometrium, altered peritoneal environment, reduced immune surveillance or increased angiogenic capacity. Moreover, retrograde menstruation does not explain the occurrence of endometriosis in extra pelvic locations. Another puzzling issue regarding the theory of retrograde menstruation is the discovery of endometriotic implants in men undergoing estrogen therapy for prostate cancer. It is well documented that the main trophic factor in endometriosis is estrogen; therefore it may well be possible that estrogen exposure plays a significant role in the development of the disease [**13**]. Coelomic Metaplasia In response to the limitations of retrograde menstruation theory, Gruenwald suggested, in 1942, that mesothelial cells of any organ, including those of the pelvic cavity, in particular the ovary, could undergo a differentiation into the functional endometrium; this became known as the theory of coelomic metaplasia and has since gathered support from several authors [**14**]. The process by which coelomic metaplasia occurs remains speculative. It may be admitted that steroid hormones or exogenous compounds induce the differentiation of normal mesothelial cells into endometriotic cells. The validation of this theory rests on its ability to explain how amenorrheic women and men undergoing hormone therapy occasionally present with endometriosis. Additional support for this theory is the prediction that endometriosis may be found anywhere where mesothelium is present. Findings of endometriosis in the pleural cavity, diaphragm, brain and several other organs, therefore add credibility to the theory of coelomic metaplasia. However, it must be noted that metastasis of endometrial cells through the circulatory or lymphatic system may also account for the presence of endometriosis at these extra-pelvic sites [**15**]. Immune System Abnormalities It has long been recognized that patients with endometriosis display alterations in the immunologic response. It is thought that defective immunosurveillance may decrease the clearance of any refluxed menstrual debris, allowing the persistence of ectopic endometrial cells within the pelvic cavity. Additionally, it is also thought that the observed abnormal immune response could promote the persistence and growth of ectopic endometrial cells. An increased concentration of macrophages has been reported in the peritoneal fluid (PF) of women with endometriosis. Macrophages are involved in recognizing foreign and damaged cells in the peritoneal cavity; once recognized, these cells are processed by macrophages for presentation to T lymphocytes. However, in patients with an endometriosis malfunction of the peritoneal macrophages, they are induced to secrete growth factors and cytokines that may promote the survival of ectopic endometrial cells. Similarly, an alteration of the production of cytokines from T helper lymphocytes may induce changes in the consistency of the peritoneal fluid allowing a favorable environment for ectopic endometrial tissue proliferation. The regulation and activation of macrophages and lymphocytes is dependent on a fine balance of cytokine expression, a balance that is upset in endometriosis [16,17]. The Genetics of Endometriosis One approach to study the underlying biological pathways leading to a complex disease such as endometriosis (the development of which is determined by multiple genetic and environmental factors) is to study the effect of genetic variants on disease causation. Endometriosis is recognized as a heritable disease due to the finding that it is significantly more common in the first degree relatives of women with the disease. The involvement of genetic factors in the development of endometriosis is supported by different studies. A study of monozygotic twins reported that endometriosis was concurrent in 14 out of 16 twin sets, with further twin studies confirming the likelihood of endometriosis arising more frequently in close relatives [**18,19**]. Studies have also shown an increased concordance in monozygotic twins when compared to dizygotic twins; in the largest such study carried out to date among Australian women, 3,096 female twins concluded that about 51% of the variation in endometriosis risk is heritable [**19**]. The heritable nature of endometriosis has prompted many to investigate which gene or sets of genes are responsible for the disease. To date, many deregulated genes have been identified in endometriotic cells with a wide variety of functions including apoptosis, cell cycle regulation, vascularization, immune system regulation and cell adhesion [**20,22**]. Some authors have linked certain genetic polymorphisms to endometriosis; however, efforts to identify genetic polymorphisms consistent across cultural and ethnic backgrounds have also failed [**23**]. Linkage studies in pedigrees have found some regions of interest, which are likely to harbor variants implicated in familial endometriosis; however, due to the methodology, the regions identified are large and contain many genes of potential interest. These regions require further investigations to elucidate the susceptibility variants. For example a multi-center study between England and Australia which examined any regions of chromosomal linkage in sister paired endometriosis sufferers, has however narrowed down the region of interest. The results of this study identified that chromosome region 10q26 is significantly associated with endometriosis [**24**] and more recent studies have indicated that region 7p15.2 is significantly associated with endometriosis [**24**]. Unfortunately, identifying the exact gene/s responsible for the heritable nature of endometriosis has failed so far. In addition to human studies, familial aggregation of non-induced endometriosis has also been shown in nonhuman primates such as the rhesus macaque [**25,26**]. There is mounting evidence for genetic variants contributing to endometriosis susceptibility. Complimentary research in animal models, particularly nonhuman primate models such as the rhesus macaque and the baboon, which can develop endometriosis spontaneously, should help to further elucidate the genetics of this complex condition. More modern thinking has led to implications for the relatively new field of epigenetics in the origin and development of endometriosis. Epigenetics concerns heritable changes to gene expression that can be influenced by environmental factors but are not the result of changes to the DNA code. Examples of epigenetic mechanisms include DNA methylation, loss of imprinting and gene regulation by microRNAs. Epigenetics has revolutionized the understanding of other complex multifactorial diseases such as cancer [**27,28**] and recently it has emerged that epigenetic mechanisms likely play a significant role in the origin and progression of endometriosis [**29**]. Environmental/Lifestyle Factors and Endometriosis Epidemiological data regarding endometriosis is currently limited, but the few studies that have been undertaken suggest that lifestyle and dietary factors may be associated with susceptibility to developing endometriosis. The results of these epidemiological studies found that a diet high in fruit and vegetables and low in meat products was protective against developing endometriosis. Additionally, women with few or no children and low body mass index (BMI) were at a higher risk of developing endometriosis [**30,31**]. Other authors have suggested that exposure to synthetic compounds such as dioxin and other polychlorinated biphenyls (PCBs) could lead to the development of endometriosis due to their effects as endocrine disruptors. Dioxin is a byproduct of the chlorine bleaching process used in the wood pulp processing industry; this also includes the manufacture of tampons which is thought to be a major source of dioxin exposure in women. However, the associations with dioxin are mainly based on animal data. Human data on dioxin exposure and endometriosis risk is scant and in some cases contradictory [**32,33**]. For example, a study reported that the incidence of deeply infiltrating endometriosis in Belgium, reportedly the highest in the world, correlates with high dioxin exposure through breast milk [**34**]. However, another study assessed massive dioxin exposure from the Seveso incident in Italy during the summer of 1976, whereby a chemical manufacturing plant accidentally released 1Kg of dioxin into the atmosphere, showering the neighboring residential areas. Although extremely high levels of dioxin contamination were found in soil and water samples, no significant increase in endometriosis incidence were observed, even after a 26 year follow up study. Despite the reported increased serum dioxin levels and increased serum levels of bisphenols observed in endometriosis patients, a conclusive association between environmental toxicant exposure and increased risk of developing endometriosis has yet to be established [**35,36**]. Symptomatology and diagnosis Despite its prevalence, this disease remains poorly understood and current studies have proved that there is no relationship between the extent of the disease and its symptomatology. While an important proportion of women with this condition may be asymptomatic, patients with endometriosis often manifest severe dysmenorrhea, non-cyclical chronic pelvic pain, dysfunctional uterine bleeding, infertility, dyspareunia, painful defecation during menstruation, urinary tract symptoms, and gastrointestinal symptoms [**37**]. However, there was a retrospective study that included 225 women with pelvic pain symptoms and DIE that concluded the next: there is an association between the frequency of severe dysmenorrhea and the presence of adhesions in the Douglas pouch, the frequency of dyspareunia is correlated with the involvement of the uterosacral ligaments, the frequency of non-cyclical chronic pelvic pain was higher when DIE involved the bowel, while painful defecation during menstruation was more frequent when DIE involved the vagina. In conclusion, this study gave evidence of a correlation between locations of DIE lesions and symptomatology [**38**]. As it is usually diagnosed in advanced stages, endometriosis implies a high rate of morbidity for this disease. A diagnosis delay of 5 to 10 years is very common in healthcare settings because the symptomatology of endometriosis shows many commonalities with a wide range of diseases [**39**]. For now, endometriosis can only be reliably diagnosed by laparoscopy and biopsy for histological confirmation of suspicious lesions. Subsequently, laparoscopy is the standard technique for the inspection of the pelvis, and for reaching to a definitive diagnosis, as well as for simultaneous treatment. Additional tools are needed for a non-invasive diagnostic and classification. The measurement of serum CA125 concentrations has no value as a diagnostic tool, therefore, for now, there is no blood test available for the diagnosis of endometriosis [**40**]. Riley and co. studied the concentrations of CA125, which they assumed that could be significantly higher in women with moderate or severe endometriosis, and normal in women with minimal or mild disease, but the lack of sensitivity and specificity regarding a cut-off value for this marker limited is its diagnostic and clinical applicability [**41**]. Treatment Up to this point, there is no very successful option for the treatment of endometriosis. Therapies are divided into two types of treatment, pharmacological therapies that aim to inhibit the growth of the endometriotic implants, and surgical therapies which try to remove or destroy the endometriotic implants [**6**]. It seems that for fertility purposes, surgical treatment followed by ART is the most beneficial available way of dealing with endometriosis, while the medical treatment is the best way to manage pain symptoms. For many decades, the surgical removal of endometriotic lesions was the primary basis for the management of endometriosis and radical removal of ectopic lesions is still the preferred way for most surgeons when dealing with endometriosis. It seems that a combined medical and conservative surgical approach is beneficial for most women with endometriosis associated pelvic pain. The patient’s own preference and plans for her fertility are more important in controlling the management plans. Medical treatment Endometriosis is an estrogen dependent disorder [**42**]. In consequence, current medical therapies are focused on reducing circulating estrogen levels. This is usually achieved by down regulating ovarian production of steroid hormones (estradiol). The effectiveness of medical treatment alone is not completely satisfactory as the evidence showed that endometriotic implants on patients who underwent gonadotropin releasing hormone (GnRH) agonists will have some persistent residual disease. Combined oral contraceptive pills (COCPs) This therapy is commonly prescribed to women with endometriosis. It consists of a combination of ethinyl estradiol and a progestin and induces a "pseudo pregnancy" state [**43**]. Although data regarding the mechanisms of action of COCPs is sparse, the initial investigation suggests they suppress proliferation and induce the apoptosis of endometrial cells. The clinical observation of the obvious reduction of endometriosis associated pain during pregnancy gave birth to the concept of treating patients with a pseudo pregnancy regime by decreasing the effect of ovarian hormones on the endometrium. Combinations of high-dose estrogens and progestogens were used at first, then progestogens alone. Modern low-dose COCPs are used in clinical practice without much high level evidence of their effectiveness. The COCPs reduce menstrual flow and decidualization of the ectopic endometrium with decreased cell proliferation and increased apoptosis [**44**]. COCPs have the great advantage over other hormonal treatments in that they can be taken indefinitely and are generally easily acceptable to women than alternative hormonal treatments, which improve compliance. Current guidelines suggest that, in the absence of a diagnostic laparoscopy, COCPs empirical treatment can be given to treat pain symptoms suggestive for endometriosis [**37**]. Although, COCPs have been used for many years by clinicians for the treatment of pain related symptoms in endometriosis, only few studies have been conducted to test the efficacy of this group of medications in a proper way. [**45,47**]. Progestogens The anti-estrogenic effect of progesterone has been mimicked in an effort to create medical therapies for endometriosis [**6**]. These compounds, known as progestogens, include Medroxyprogesterone acetate (MDPA) and Norethisterone acetate (or norethindrone acetate, NETA). Oral progestogens have been used for the treatment of endometriosis and endometriosis-associated complaints for more than 40 years. Progestogens––especially non-androgenic progestogens–– are well tolerated and have only few side-effects; they can be used repeatedly or continuously over a long period of time. The drugs induce a pseudopregnancy state in which endogenous estrogen production is lowered. The mechanism of action of these drugs is thought to consist of the suppression of the estrogen receptors, leading to an endometrial decidualization and subsequent atrophy [**48**]. Additionally, these compounds have been shown to decrease the expression of matrix metalloproteinases, enzymes, which are thought to be essential for the implantation and growth of ectopic endometrial cells. Although these drugs are better tolerated than Danazol, their use still produces some side effects [**49**].This modality of treatment should be restricted to a group of women with pain symptoms and not wishing to conceive. Unfortunately, the pain symptoms usually relapse after cessation of treatment. Although, their effectiveness is well recognized, yet there are not many randomized trials to confirm that [**48**]. MDPA has been used for many years for the pain symptoms in endometriosis. In a multicenter RCT, subcutaneous DMPA was compared with leuprolide for endometriosis-associated pain. DMPA and leuprolide produced equivalent reductions in pain scores. At month 6, reductions in total hip and lumbar spine bone mass density (BMD) were significantly less with MDPA-SC 104 versus leuprolide, findings confirmed by other studies. MDPA is an effective, safe and cost effective alternative for the treatment of symptomatic endometriosis. However, because of its effect on ovulation it is not a good candidate for women seeking fertility. Another problem is the uterine breakthrough bleeding which may be troublesome. NETA, 10 mg was proven to be very effective in treating women with pain symptoms related to endometriosis with an overall efficacy of 94%. The continuous administration of norethisterone acetate to treat endometriosis is approved by the US FDA. Dienogest is a synthetic steroid that has been used as a progestogen in contraceptive pills and is currently being studied for its possible clinical use in the treatment of endometriosis. It exhibits highly selective binding to the progesterone receptor and has high progestational and significant antiandrogenic activity, but only moderate antigonadotrophic activity. Adverse effects associated with dienogest are the same as those expected from a progestogen (weight gain, increased blood pressure, breast tenderness and nausea). Dienogest was used for many years for the treatment of endometriosis and research demonstrated that this medication at a dose of 2 mg daily for 12 weeks was significantly more effective than placebo for reducing endometriosis associated pain [**50**]. Progestogen mini-pill These synthetic agents have been used in the management of symptomatic endometriosis both as primary therapy and as an adjunct to surgical resection. Desogestrel (75 µg/d) was used after laparoscopic surgery on women with endometriosis in favor of COCPs (ethinyl estradiol 20 µg plus desogestrel 150 µg). Desogestrel was shown to be effective, safe and cost effective. Levonorgestrel-intrauterine system (LNG-IUS) Levonorgestrel is a 19-nortestosterone that induces decidualization and acyclicity of the endometrium and endometriotic tissue. Levonorgestrel locally administered through the intrauterine system has a profound effect on the endometrium, which becomes atrophic and inactive; thus producing amenorrhea or hypomenorrhea and greatly reduces menstrual pain associated with endometriosis especially for women who do not wish to become pregnant, this device offering the possibility of at least 5 years of treatment following one single intervention [**51**]. Furthermore, LNG-IUS probably reduces recurrence or development of new endometriosis. The LNG-IUS does not provoke menopausal symptoms. The only concern in LNG-IUS is the bleeding problems that might be troublesome for some patients. Etonogestrel subdermal implants (Implanon) are an additional treatment option in women with symptoms related to pelvic endometriosis and has the potential of providing long-term treatment of endometriosis. However, women should be carefully counseled regarding menstrual changes. Recently, it has been suggested to be used in combination with LNG-IUS in cases of persistent pelvic pain due to endometriosis not responding to conventional treatments. This option should be theoretically beneficial in endometriosis, since simultaneous use of subdermal implant and LNG-IUS will provide both systemic and local progestogens [**52**]. Weak androgenic steroids Danazol, an isoxazol derivative of 17α-ethinyl testosterone was the first drug to be approved for the treatment of endometriosis in the US [**1**]. The efficacy of danazol is based on its ability to produce a high androgen and low estrogen environment (a pseudo menopause) which results in the atrophy of the endometriotic implants and thus an improvement in painful symptoms. Its anti-steroidogenic activity is known to relieve some of the symptoms associated with endometriosis, however substantial androgenic side effects, such as hair growth, mood changes and more seriously, liver damage and arterial thrombosis have been reported [**53,54**]. GNRH agonists / analogues GnRH agonists are analogs of the hypothalamic hormone GnRH. GnRH is responsible for the normal function of the ovaries by stimulating the release of follicle stimulating hormone (FSH) and luteinizing hormone (LH) from the pituitary gland. Normal menstrual cycles rely on pulsatile delivery of GnRH to the pituitary. GnRH agonists bind to pituitary receptors resulting in a shutdown of pituitary hormone secretion which in turn down regulates the ovarian production of estrogen. This down-regulation is constant and results in a profound hypoestrogenic state much like that which the body undergoes during menopause. Endometrial implants are estrogen dependent, so with this artificial menopause they subsequently regress. Starving the endometriotic cells of estrogen in combination with alterations of plasminogen activators and matrix metalloproteinases [**55**] is thought to result in endometriotic atrophy. However, endometriotic cells are known to express the aromatase enzyme, ensuring their survival independent of ovarian steroids. GnRH agonist therapy is also associated with the side effects normally presented during menopause, such as hot flashes and loss of bone density, therefore in some cases small amounts of steroid hormone are administered in what is known as "add back" therapy which appears to stem the severity of side effects without significantly affecting the relief of endometriosis associated pain. Because of the adverse effects, the use of these drugs is limited to 6-months duration. Pelvic pain associated with endometriosis was treated by the long-term administration of a tapering dose of danazol or mid/low doses of COCPs after the end of therapy with GnRH agonists. Results demonstrated that each of these therapies is a practical and efficient treatment regimen to maintain the relief of pelvic pain achieved by GnRH-a therapy, at least for a period of 12 months [**56**]. GnRH antagonists At the molecular level, GnRH antagonists interrupt the basic activation process of the GnRH receptor (GNRH-r). When GnRH binds to its receptor, the receptor dimerizes and initiates a cascade of events leading to the synthesis and secretion of the luteinizing hormone (LH) and follicle stimulating hormone (FSH). In one study on 15 patients, GnRH antagonists were found to alleviate pain symptoms of endometriosis of patients while under treatment [**57**]. The need for an oral form of GnRH antagonist led to the discovery of a small molecule, CMPD1, which binds with low nanomolar affinities to human, rat and mouse GnRH-R. Animal studies have shown that, CMPD1 is a potent, selective, orally active GnRH antagonist that may have potential application as a therapeutic agent for treating hormone-dependent cancers and diseases including endometriosis [**58**]. Other novel therapies Aromatase inhibitors have almost graduated from the pantheon of potential future therapies for endometriosis and they are now considered an acceptable therapy for endometriosis that does not respond to conventional treatments since they have shown promising preliminary results [**59**]. Drugs which inhibit the endometrial and endometriotic aromatase enzyme systems should be theoretically beneficial in endometriosis, since they reduce the local production of estrogen. The conversion of C19 steroids to estrogens occurs in a number of tissues, such as the ovary and placenta, and is catalyzed by aromatase P450. This enzymes expression has also been detected in leiomyomas and endometrial cancer and appears to be undetectable in normal endometrial and myometrial tissues. But P450 aromatase was found to be expressed in both eutopic and ectopic endometrium from patients with endometriosis. The reported over-expression of aromatase in endometriotic cells highlights how endometriotic cells appear so resilient and self-sustaining. Aromatase expression is also found to be increased in certain cancerous cell types, in particular breast cancer. Aromatase inhibiting drugs such as letrozole and anastrozole are established therapies for breast cancer and these drugs are currently undergoing a clinical trial as a possible therapy for endometriosis, with some encouraging results [**60,61**]. Moreover, a case report of five cases has shown that Letrozole administered with combined pills achieved complete regression of recurrent endometriotic cysts and pain relief in all cases. In animal models, a new selective aromatase inhibitor, fadrozole, appeared to be useful for the treatment of endometriosis. Aromatase inhibitors appear to have a promising effect on pain associated with endometriosis, however, their use is associated with the side effects of creating a dramatically hypoestrogenic environment, such as bone density loss. Selective estrogen receptors modulators Theoretically, drugs which block the estrogen receptors should interfere with endometriosis development and progression. Raloxifene is a nonsteroidal, selective estrogen receptor modulator (SERM). In a rat model for endometriosis, raloxifene was found to reduce ectopic lesions. However, in a randomized controlled study, raloxifene had significantly shortened the time to return of chronic pelvic pain associated with endometriosis. Tamoxifen is another drug that belongs to this group and has been suggested as an option for endometriosis treatment [**62**]. Selective progesterone receptors modulators Progesterone receptor modulators (PRMs) are progesterone receptor ligands. Mifepriston (RU-486) was the first of this class of drugs to be used in treating endometriosis. The drug is primarily a progesterone antagonist that can inhibit ovulation. Daily doses of this medication range from 5 to 100 mg daily. In very limited clinical trials, mifepristone has shown benefits in some patients in terms of reduced pain and regression of lesions. Other possible medical approaches The other modalities are possible candidates, such as pentoxifylline and other immunomodulators, matrix metalloproteinase inhibitors, angiogenesis inhibitors and tumor necrosis factor-alpha (TNF-α) inhibitors. Recently, the use of 5-fluorouracil [5-FU] has been investigated in cases with severe types of endometriosis with limited success. Future Therapies Due to the relatively poor efficacy of hormonal therapy for endometriosis, several other experimental therapies are currently undergoing clinical trial, such as Mifepristone, a selective progesterone receptor modulator, therapies designed to exploit the characteristics of endometriotic cells as potential drug targets such as anti-angiogenic therapy. TNP-470, endostatin, VEGF-A blocking antibodies, methoxyestradiol and anginex, have all shown as potential anti-angiogenic therapies for endometriosis in animal models [**63**]. However, drawbacks to this last approach include: reduction in fertility observed in animals and patients taking anti-angiogenic therapy for cancer and the possibility that not all the endometriotic tissue would be regressed by a single anti-angiogenic therapy owing to the heterogeneous nature of diseased tissue. Recently, Hassan et al have suggested that gene therapy may be a novel approach to the endometriosis treatment [**64**], this involving the delivery of the genetic material to diseased cells via a suitable vector in order to correct aberrant gene expression observed in diseased cells. Another approach suggested by Hassan et al was to use adenovirus vectors to deliver dominant negative mutants of the estrogen receptor (DNER) to endometriotic cells, which would greatly reduce the sensitivity of endometriotic cells to estrogen and thus, atrophy their growth. Early results from this therapy have been encouraging, indicating increased cell death in endometriotic cells [**65**]. Such a treatment regime would confer advantages to the patient. Gene therapy may also allow targeted therapeutics to specific cell types and act at the cellular level greatly reducing unwanted side effects. However, gene therapy is still in its infancy and suffers certain drawbacks such as rejection of the viral vectors by the host, short half-life of the therapeutic DNA, and as only one gene can be corrected at a time, at present it is unsuitable for multifactorial diseases like endometriosis. Surgical management of endometriosis If medical therapy proves unsuccessful or the stage of endometriosis is considered too advanced, then the surgical intervention is often the only remaining option. However, the appropriate treatment varies significantly depending on the woman’s age, parity and the nature of the symptoms. Surgery falls into two main categories, conservative surgery which can usually be performed via laparoscopy and radical surgery which normally involves the partial or entire removal of the affected organ. The aim of the surgical management is to remove the visible areas of endometriosis and restore the anatomy by division of adhesions. The severity of pain symptoms in minimal peritoneal disease were found not to be correlated with findings at laparoscopy. Surgical approaches like resection of peritoneal endometriosis; or monopolar electrocoagulation/ablation may be used. In the early stages, surgical treatment seems to reduce pain symptoms as effectively as by drug therapy. Laparotomy (open surgery) was considered the standard method for surgical therapy of endometriosis before the recent advances in laparoscopic techniques. During laparotomy, surgeons perform a proper manual exploration of retroperitoneal spaces, examination of bowels, and delicate handling of deep lesions. In severe endometriosis it was found that laparotomy and laparoscopy are equal in treating pain and infertility problems, yet there is a trend towards a higher pregnancy rate and lower dyspareunia recurrence rate after laparotomy, compared with laparoscopy. It seems that laparotomy will still have a place if the laparoscopic skills of the treating surgeon are not enough to deal with the disease [**66**]. Laparoscopic uterine nerve ablation (LUNA) and presacral neurectomy were also suggested as a treatment of pain symptoms. LUNA was found beneficial for dysmenorrhea not associated with endometriosis, while presacral neurectomy was found to reduce pain symptoms in endometriosis without significant side effects [**66**]. Conservative surgery - Surgical Excision/Ablation of Endometriosis Jansen and Russell have shown that peritoneum which looks completely normal does not contain histological features of endometriosis, therefore, excision of all abnormally looking peritoneum, deep nodules and ovarian lesions should remove the disease. Yet a downside of this approach is the risk for future adhesion formation. Additionally, the complete excision of an endometriotic implant can be difficult. The degree to which endometriotic implants cause pain is strongly related to the nature of the implant. It has been reported that deeply infiltrated endometriotic implants are most commonly associated with pain. Superficial implants on the other hand are often associated with minimal symptoms [**67**]. The excision of the endometriotic implant can be performed by using a variety of techniques but they all involve the cutting away of endometriotic tissue from the healthy tissue. There are few well designed studies to assess the success of the excisional therapy for the treatment of endometriosis associated symptoms therefore it is hard to assess the benefits of this procedure over others. Koninckx et al reported that despite an aggressive surgical approach, the total removal of the implant could not be achieved more that 90% of the time [**67**], and even then, the complete the removal of the implant was associated with serious complication in a quarter of cases. Some surgeons forgo the use of excisional therapy in favor of ablative destruction of the endometriotic implant. This can be performed via a variety of techniques such as electrocautery or laser vaporization. There are many reports on the use of ablation for the management of the symptoms of endometriosis. The overall conclusions that can be drawn from these studies indicate that surgical ablation has around 60% success rate after 2 years (this means that 60% of the patients were pain free after 2 years). However, the pain and the individual’s response to pain differ, affecting the conclusion that can be drawn from these studies. In the resection technique, the peritoneum is incised near the lesion by using a monopolar electrode and is dissected bluntly, separating healthy tissue from endometriotic tissue. Resection seems to be more efficient than diathermy coagulation, yet, it is more difficult, increases the time of the operation, and the costs. Operative laparoscopy should be the first choice for the management of ovarian endometrioma whenever possible and seems to be effective in alleviating pain associated with ovarian endometriomas [**68**]. The surgical laparoscopic management of ovarian endometriomas can involve the stripping of the cyst lining or laser vaporization of the internal wall of the cyst. If laser is used, the depth of this vaporization may be superficial and only the glandular epithelium and the adjacent stroma have to be vaporized. Medical therapy alone has not generally been effective in reducing endometrioma size and formed adhesions [**68**]. Radical surgery If the management of endometriosis is not achievable by medical and minor surgical intervention, radical surgical options may have to be explored. Often, this involves the partial or complete removal of the affected organ. The most common procedures include hysterectomy and oophorectomy, although the removal of the ovaries would require premenopausal patients to undergo hormone replacement therapy so as to lessen the symptoms of estrogen deprivation. This option can be offered to women with chronic pelvic pain with endometriosis who completed their families and underwent other medical and or conservative surgical treatments. However, this approach may not always alleviate pain symptoms especially in deep endometriotic disease. A seven years follow up study of 240 women who underwent surgery reported the following findings on the effect of various surgical procedures for the relief of endometriosis associated pain based on the need for further surgery [**69**]. Firstly it would appear that the surgical excision alone frequently requires further surgery suggesting a high rate of disease recurrence. A total hysterectomy with oophorectomy was the most successful long term surgery. This is most likely due to the removal of the source of potential refluxed endometrium (the uterus) and the source of mitogenic steroid hormones (the ovaries). Therefore, this also gives support to the retrograde menstruation theory; as it would appear that removing the anatomical features necessary for the retrograde menstruation drastically reduce the recurrence of endometriosis. However, this study did not address issues such as what medical therapy, if any, the patients selected for this study were taking post-operatively, a factor which may influence the recurrence of the disease [**69**].
